# The Medical Treatment of Cholelithiasis

**Published:** 1914-01

**Authors:** 


THE MEDICAL TREATMENT OF CHOLELITHIASIS. H. B. Anderson, M.D., L.R.C.P., (Lond.), M.R.C.S. (Eng.), Associate professor of Clinical Medicine in the University of Toronto, Toronto,

Canada. *Monthly Cyclopedia and Medical Bulletin,* November 1913. Following the teaching of Naunyn, Kehr, Aschoff, and other German investigators, a much broader view of the whole pathological process is being taken in the formation of gall-stones is regarded as a mere incident of a disease in which *bile-stasis, infection,* and *more or less widespread inflammatory manifestations* are of *primary* importance, and against which treatment, whether medical or surgical, must be directed. Attention is being directed more to *biliary stasis, infection,* and *inflammatory manifestations,* rather than focused on the *secondary result*—the calculi. Kehr considers the calculi in inflammation of the biliary passages in the same light as the fecolith appendicitis—a product of the inflammation. Since Aschoff has shown that *non-inflammatory cholesterin calculi* may occur, due to bile-stasis and certain metabolic disturbances, as in pregnacy, Naunyn's view as to the *invariable* presence of infection is no longer tenable on pathological grounds, though clinically the fact remains that these cases are unimportant so long as no infection occurs.

All these authorities believe that medical measures may be effective in relieving the inflammatory trouble in many instances, and in producing a virtual cure.

The belief that gall-stones might be dissolved was long held by many physicians. Naunyn thought this might occur in rare instances. This view has been largely discarded, but some recent investigations by Hansemann have reopened the question. He has apparently proved by experiments *in vitro,* and by transferring gall-stones from human beings to dogs, that gall-stones are soluble in normal bile, particularly stones composed largely of cholesterin. He believes, therefore, that if by treatment the catarrhal conditions of the bile-passages can be cured, inflammatory products removed, and the bile restored to its normal condition, the stones will be dissolved spontaneously. Treatment, according to him, should be undertaken with this aim in view, rather than with a view to direct action on the gall-stones. The article is accompanied by a number of illustrations, showing sets of gall-stones in various stages of being dissolved, the research reported establishing once more that normal bile under normal conditions does not permit concrements to develop, and will dissolve in time those already formed. The peculiar shape of the gall-stones found at operation in many cases is due to their being partly dissolved. Hansemann's work, if confirmed, will obviously have an important bearing on treatment.

In proportion to the general incidence of gall-stones in from 5 to 10 per cent. of autopsies, the occurrence of many of the most serious complications, such as cancer, gangrenous cholecystitis, gall-stone ileus, etc., is so infrequent that the danger of their development in a given case is too remote to constitute *in itself* a reason for operation as a *general procedure.* Is it correct to assume that recourse to early operation would appreciably reduce the general mortality by forestalling these rarer complications, when in the majority of all cases gall-stones are latent, or at least do not produce symptoms sufficiently definite to permit of a diagnosis, and especially when the course of the disease will give timely warning in most of the diagnosable cases and direct them to the surgeon? I believe that many clinicians will agree with Kehr, that it is fortunate that we are unable, by the X-rays or other means, to recognize the quiescent cases, if the presence of gall-stones *per se* is to be taken as an indication for their removal.

But again, it is claimed that operative cures are permanent, whereas medical cures are merely palliative. This statement is scarcely in accord with our knowledge of the pathology of the disease or our clinical experience. After operations the bile-stasis, infection and inflammatory changes must subside, and the underlying causative factors be removed, before the patient can be regarded as cured. If the gall-bladder is not removed, and if the causes underlying the original infection which produced the gall-stones remain or recur, on what grounds have we a right to claim that the stones will not form again, as in the first instance? Operative procedures have not been in vogue sufficient length of time to determine definitely how frequently recurrences may take place, but that they do occasionally is well known. I have at present a case under observation with symptoms of recurrence six years after operation. Gerster, in a paper on "Unsuccessful Surgery in Disorders of Bile-ducts," reports 11 per cent. of relapses in 57 operative cases, and quotes Ochsner's failures in 15 per cent. in *calculous* and 54 per cent. in *non-calculous* cases. It is therefore apparent that the claims of *certainty of cure* and *permanency of result* after operation cannot be accepted without qualification.

The danger from operation, even in the most skilled hands, is not to be overlooked. The mortality in Mayo's series of 4,000 cases was 2.57 per cent., and in the cases collected by Bland-Sutton from the English hospitals in 1905, 17.7 per cent., the results varying with the conditions calling for relief and the experience and skill of the operator. Nor must we confound recoveries from operation with the cure of the patient's ills. As with medical cures, only time can show the permanency of relief. Gerster and others believe that drainage after operation is essential for the relief of the infection, and undoubtedly this is the case; but it has been found that in non-calculous cholecystitis, where the infection and its sequences alone are to be dealt with, operation frequently fails to give relief.

